# Radiographic 2D:4D index in females: no relation to anthropometric, behavioural, nutritional, health-related, occupational or fertility variables

**DOI:** 10.1186/1477-5751-5-12

**Published:** 2006-08-25

**Authors:** Tapio Vehmas, Svetlana Solovieva, Päivi Leino-Arjas

**Affiliations:** 1 The Finnish Institute of Occupational Health, Topeliuksenkatu 41 a A, FIN-00250 Helsinki, Finland

## Abstract

**Background:**

The ratio of index finger to ring finger length (2D:4D index) may be an indicator of gonadal hormone exposure, because the differentiation of gonads, fingers and toes is influenced by the same HOXA and HOHD genes. Some previous studies have found significant associations between the 2D:4D index and sexual, psychological or behavioural variables. We studied the usability of the radiographic 2D:4D index as a potential predictor of several features in a large female sample.

**Methods:**

271 female dentists and 219 teachers (age 45 – 63 years) had their hands radiographed and their right 2nd and 4th fingers measured from the base of the bony proximal phalanxes to the tip of the distal phalanxes to define the radiographic 2D:4D index. The study subjects were classified into two distinctly separate clusters (by using cluster analysis with the K-means algorithm) in each of the following dimensions: anthropometric (including four items), behavioural (five items), nutritional (five items), health-related (seven items), occupational (Karasek job control and job demand scores) and fertility (four items).

**Results:**

The radiographic 2D:4D index ranged from 0.845 to 0.981 (mean 0.925, SD 0.021). The intraclass correlation between three radiographers' measurements (31 cases) was 0.971. No differences concerning the 2D:4D index were found between clusters 1 and 2 in any studied dimension, nor did any of the items in clusters have relations with the 2D:4D index when tested separately with bivariate tests.

**Conclusion:**

Despite the ideal set-up of the measuring possibilities in a relatively large radiographic material the variables currently studied were not dependent on the length of finger bones. It can therefore be questioned whether any real associations between the bony 2D:4D index in adult life and (direct or indirect) hormone dependent effects exist. There may be a publication bias explaining that mostly positive findings have been the previously reported. However, the associations of the 2D:4D index with various features, if present, may be related to the soft parts of fingers rather than to the length of bones.

## Background

The ratio of second to fourth finger length (2D:4D index) in the right hand has been intensively studied during the recent few years. This index is lower in men than in women and varies also within sexes. There is no significant change in this index during childhood [1]. The index is probably established in uterus and has first been shown to be a predictor for sperm numbers and the concentration of testosterone, luteinizing hormone and oestrogen [2]. The Homeobox or HOX genes A and D influence the differentiation of fingers, toes and gonads, the products of which (such as testosterone) may therefore be reflected in the morphology of fingers and toes [3]. Thereafter, the main hypothesis has been that low index (short second finger as compared to the fourth finger) is associated with a masculine type of behaviour and vice versa. In the Bem Sex Role Inventory test it was found that low 2D:4D ratios associated significantly with higher, masculinized bias scores indicating that the 2D:4D ratio predicts the self-reported sex-role identity in females [4]. In right-handed children, a high 2D:4D index correlated with an improved right-hand skill and a low 2D:4D index correlated with an enhanced left-hand skill [5]. High aggression scores were associated with masculinized (low) right hand 2D:4D indices in females [6]. The examination marks of male university students have been shown to correlate positively with the finger length ratios [7]. Several studies with somewhat different results have also been published on the association between the 2D:4D index and sexual orientation [8]. Among 40 healthy adult female students studied by magnetic resonance imaging it was found that the 2D:4D ratio was associated with an asymmetry in the hippocampal sub-regions [9]. It thus seems that this index is based on an anatomic alteration in brain and may thus have a profound effect on human behaviour.

The 2D:4D index has usually been measured either directly from hands or from photocopies, which may, however, yield lower indices than direct measurements [10]. These investigators concluded that measurements made by using different techniques should not be combined within one study nor should they be used together in comparative studies and that finger length differences between direct measurements and photocopies could result from the shapes of fat-pads at the tips of the fingers and these may be dependent on sex and sexual orientation.

We have previously conducted a project among a large sample of female dentists and teachers with the primary aim to study occupational and other factors related to radiographic hand osteoarthritis [11] and cortical bone mass [12]. In this secondary study we have collected information regarding anthropometric, behavioural, nutritional, occupational, health-related and fertility factors aiming to work out whether they are related to the 2D:4D index. We have radiographed the hands of all study subjects and have retrospectively measured the radiographic 2D:4D index of the right hand. We use this radiographic index to study its association with the above mentioned factors.

## Methods

### Study subjects

The potential participants were identified from trade union registers (Finnish Dental Association and Finnish Teachers Trade Union). Questionnaires were randomly submitted to 436 female dentists and 436 female teachers by using the place of residence as an inclusion criterion (Helsinki or its neighbouring areas). Of those, 295 dentists and 248 teachers participated in the examinations including clinical visit (the measurement of weight, hand size and grip strength and radiography of both hands). Body mass index (BMI, kg/m^2^) was calculated as weight (kg)/height (m)^2^. Due to missing radiographs and technical errors in radiographs (usually the non-visibility of finger tips) only 490 cases could be analyzed (271 dentists and 219 teachers, age 45 – 63 years, mean 54 years).

Each participant filled in a 10 page questionnaire on background, occupational and health-related variables. Based on data thus formed the independent variables were classified as indicated below:

1) anthropometric variables (body mass index: current and at the age of 25 years, reported hand size [small, medium, large] and handedness [right hand/left of both hands]),

2) behavioural variables (hand loading hobbies such as instrument playing, cooking, cleaning, gardening or handiwork [hours/week] and hand loading leisure time physical activities such as loading handiwork, construction work, hand loading sports [hours/week], household chores [hours/week], alcohol consumption [drinks/week] and smoking history [never smokers and ever smokers, the latter including current smokers and ex-smokers]),

3) nutritional variables (coffee consumption [cups/day], milk consumption [dl/day], daily calcium intake [mg] calculated as the sum of announced calcium intake from dairy products and from vitamin supplements, alcohol consumption [drinks/week] and fruit and vegetable consumption [several times per day, daily, not every day]),

4) work-related variables (an 11 item job content questionnaire [13], out of which two sum scores were constructed: job control and job demand) and

5) health-related variables (finger pain during the past 30 days [sum score of individual joints], hand radiographic osteoarthritis [sum score of individual joints], metacarpal index [dominant hand], measured grip strength [dominant hand], hormone replacement therapy at menopause [years], use of hormonal contraceptives [years], a 12-item general health questionnaire [sum score]

6) fertility variables (marital status [married, not married], number of pregnancies, use of hormonal contraceptives [ever, never], use of hormones [ever, never].

The study plan was accepted at the local ethics committee and the subjects gave their informed consent to study participation.

### Radiography and measurement of the 2D:4D index

**Figure 1:**
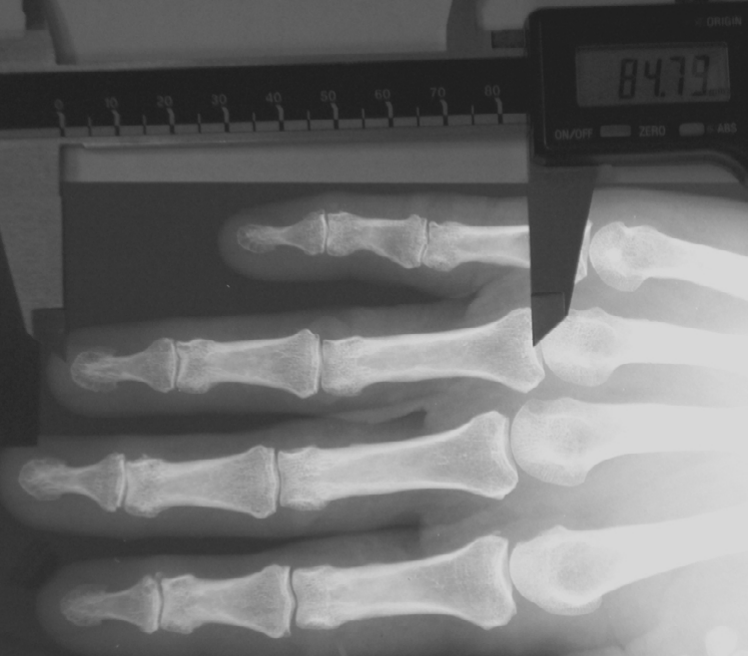
Calliper measurement of bony finger length (actual measurement situation is more accurate than in this image).

Both hands were radiographed with Siemens analogue equipment (Siemens Munich, Germany) and radiographs were inspected at lighted view boxes. After mutual training the 2D:4D index of the right hand was measured by three radiographers and each of them managed about one third of the cases. They first measured separately a set of 31 cases to work out their interobserver agreement. The distances from the bases of the second and fourth proximal phalanxes to the tips of the corresponding distal phalanxes were measured with Mitutoyo CD-15CP calliper scaled at 0.01 mm (Mitutoyo corp, Japan) (Fig. 1). The calliper was calibrated before every measurement. The radiographers worked blinded to any clinical data of the subjects.

Osteoarthritis (OA) in each finger joint was graded by a radiologist as previously described (0 = normal, 1 = doubtful OA, 2 = mild OA, 3 = moderate OA, 4 = severe OA) by using reference images [11]. The OA sum score was the added up score in each finger joint. Metacarpal cortical index was used as an estimate of the cortical bone mass and after the radiologist's measurements it was calculated as the ratio (D_out _– D_inn_): D_out_, where D_out _and D_inn _stand for the outer and inner cortical diameters of the right second metacarpal at the thinnest point of its diaphysis [12].

### Statistical methods

**Figure 2:**
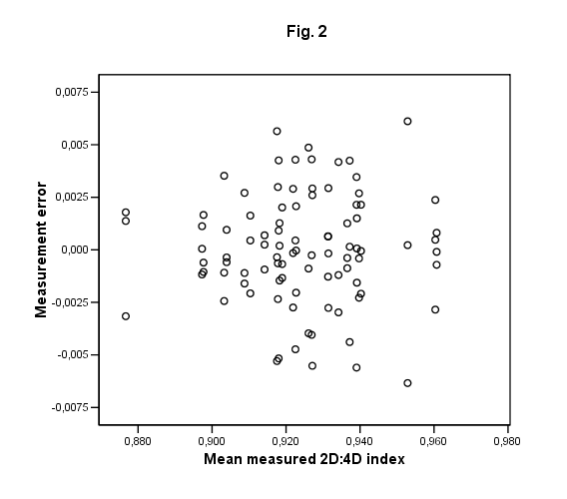
Accuracy of three observers' measurements. Error of single measurements (y-axis) plotted on the mean value of these observers (Bland-Altman plot). The mean absolute error is 0.002 (or 0.22 % out of the mean index of 0.924).

The agreement between the radiographers' measurements was assessed by computing the single measures intraclass correlation and constructing an "error proportional to the mean" plot [[14], Fig. 2].

The 2D:4D indices between categorical variables were tested either with the t-test (two group comparisons) or with the analysis of variance (more than two groups). Osteoarthritis causes narrowing of joint spaces and thus shortens fingers. Because such changes were common among our study subjects adjusted p-values were computed. The above crude analysis was supplemented by the analysis of covariance adjusted for the osteoarthritis score of the of the second and fourth finger proximal and distal interphalangeal joints. The 2D:4D indices were correlated with continuous variables with the Pearson's correlation. These analyses were supplemented by partial correlations where the above relation was controlled for the osteoarthritis score.

To avoid the potential multiple comparison problems related to testing many variables separately in terms of the 2D:4D index, we used also an alternative method, the cluster analysis. This is an effective method for defining distinct subgroups of individuals with similar features. Homogeneous (based on certain set of variables) subgroups of study participants were empirically defined by using cluster analysis with the K-means algorithm. Based on the set of variables the following six cluster classifications were formed: 1) anthropometric clusters, 2) behavioural clusters, 3) nutritional clusters, 4) work-related clusters, 5) health- related clusters and 6) fertility clusters. The assignment of a case to a cluster is dependent on the centre closest to the case in Euclidian distance [15]. The differences between the clusters were compared by using the one-way analysis of variance (continues variables) and the chi-square test (categorical variables).

SPSS software was used. P-values < 0.05 were considered statistically significant.

## Results

The symmetrically distributed 2D:4D index ranged from 0.845 to 0.981 (mean 0.925, SD 0.021). The intraclass correlation between the radiographers' measurements was 0.971. No categorical individual variables showed any significant differences in their mean 2D:4D indices nor did the 2D:4D indices show any significant correlations with continuous variables (Tables 1, 2). The effect of adjusting the analyses for finger osteoarthritis changes was little.

**Table 1:** 2D:4D index vs. background and occupational variables: relation between the 2D:4D index and categorical variables (t-test or ANOVA) and between 2D:4D index and continuous variables (Pearson's correlation).

**Variable (- categories)**	**Number**	**2D:4D**	**Correlation coefficient**	**p-value (crude)**	**p-value (adjusted)**
**Background data**					
Age	490		0.008	0.868	0.811
Stature	490		0.009	0.847	0.927
Body mass index (kg/m^2^)	489		-0.050	0.273	0.314
Hand dominancy				0.898	0.949
-right hand	451	0.925			
-left or both hands	39	0.924			
Marital status				0.374	0.341
-single	42	0.924			
-married	366	0.924			
-divorced	66	0.928			
-widow	16	0.932			
Number of pregnancies	489		0.006	0.888	0.942
Smoked years (number)	490		-0.019	0.668	0.655
Alcohol consumption index			-0.039	0.383	0.367
**Occupational data**					
Occupation				0.679	0.693
-dentists	271	0.924			
-teachers	219	0.925			
Dentists' education				0.663	0.870
-general practitioner	217	0.925			
-specialist	54	0.924			
Working status				0.099	0.138
-currently working	462	0.925			
-retired	10	0.911			
-stopped to work	18	0.927			
Part time retirement				0.728	0.941
-no	468	0.925			
-yes	22	0.923			
Weekly working hours	490		0.018	0.684	0.689
Karasek score (Karasek et al. 1998)					
-job control	486		-0.038	0.404	0.350
-job demand	486		-0.037	0.421	0.414

Adjusted p-values controlled for the shortening effect of osteoarthrotic changes in the second and fourth fingers (analysis of covariance or partial correlation). Number = number of patients in uncontrolled analysis

**Table 2:** 2D:4D index vs. health variables: relation between the 2D:4D index and categorical variables (t-test or ANOVA) and between 2D:4D index and continuous variables (Pearson's correlation).

**Variable (- categories)**	**Number**	**2D:4D**	**Pearson coefficient**	**p-value (crude)**	**p-value (adjusted)**
Self-reported cardiovascular disease				0.954	0.972
-no	437	0.925			
-yes	53	0.925			
Self-reported breast cancer				0.721	0.797
-no	482	0.925			
-yes	8	0.922			
Self-reported diabetes				0.265	0.185
-no	484	0.925			
-yes	6	0.911			
Self-reported hyperlipidaemia				0.855	0.800
-no	477	0.925			
-yes	13	0.924			
Total experienced finger pain during the last 30 days	490		-0.066	0.147	0.147
Hand radiographic osteoarthritis sum score	490		0.018	0.694	
Metacarpal index, dominant hand	488		-0.073	0.109	0.120
Grip strength, dominant hand	488		-0.034	0.452	0.457
Hormone replacement therapy (years)	255		-0.030	0.636	0.577
12-item general health questionnaire sum score	490		-0.005	0.909	0.954

Adjusted p-values controlled for the shortening effect of osteoarthrotic changes in the second and fourth fingers (analysis of covariance or partial correlation). Number = number of patients in uncontrolled analysis

### Results of cluster analyses

We assumed that aggregation of several items would be a more effective method to evaluate the discriminating effect of the radiographic 2D:4D index than the individual items. Altogether six classification procedures with two identified clusters per each procedure were performed. Women in the anthropometric cluster 2 had statistically significantly lower BMI (at the age of 25 years and current) and smaller hands as compared with those in the anthropometric cluster 1. Women in the behavioural cluster 2 spent statistically significantly more time on leisure time physical activities, other hobbies and household chores and drank less alcohol than those in the behavioural cluster 1. Daily calcium intake, milk and coffee consumption was statistically significantly higher among women in the nutritional cluster 2 than in the cluster 1. Women in the work-related cluster 1 characterized their job as more demanding and with a higher level of control than women in the work-related cluster 2. Women in the health-related cluster 2 had a better health (less finger pain score and hand OA score, a lower general health questionnaire [GHQ, 16] score) and a higher grip strength in the dominant hand than women in the health-related cluster 1. Women in the fertility cluster 1 had had more pregnancies and used less hormonal replacement therapy and also hormonal contraception than women in cluster 2, although the last difference was not statistically different.

We explored first the relationships between the index and the empirically derived clusters 1 and 2 in the six dimensions (Table 3). No statistically significant relations were found with all p-values being greater than 0.2.

**Table 3:** 2D:4D index vs. empirically derived clusters (t-test). Adjusted p-values controlled for the shortening effect of osteoarthrotic changes in the second and fourth fingers (analysis of covariance)

**Empirical classification based on**		**Number**	**2D:4D**	**p-value (crude)**	**p-value (adjusted)**
**Anthropometric parameters**				0.273	0.258
	Cluster 1	168	0.923		
	Cluster 2	320	0.926		
**Behavioural variables**				0.413	0.415
	Cluster 1	349	0.925		
	Cluster 2	139	0.924		
**Nutritional variables**				0.420	0.448
	Cluster 1	320	0.924		
	Cluster 2	170	0.926		
**Work-related variables**				0.301	0.275
	Cluster 1	258	0.926		
	Cluster 2	228	0.924		
**Health-related variables**				0.972	0.789
	Cluster 1	83	0.924		
	Cluster 2	397	0.925		
**Fertility variables**				0.55	0.55
	Cluster 1	57	0.927		
	Cluster 2	485	0.925		

## Discussion

Unlike most previous investigators, who used direct measurements or photocopies of hands, we found no significant relations between the 2D:4D index and either the tested clusters or single variables.

**Figure 3:**
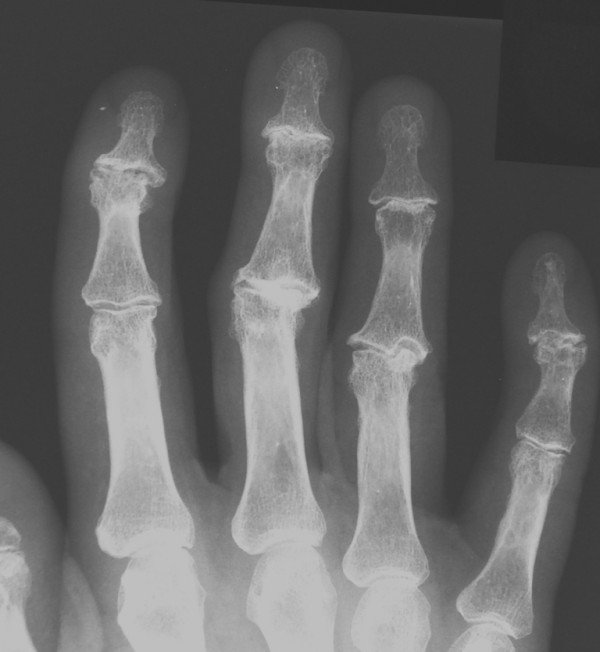
Osteoarthritis makes joint spaces narrow and thus biases finger length measurements.

By using radiographs true bony lengths may be measured and wrinkles in soft tissue or differences in nail characteristics do not disturb the measurements. Radiographic dimensions may be less vulnerable to differences in hand positioning. Also, osteoarthritis in finger joints, which is common in middle-aged persons and leads to shortening of fingers due to joint space narrowing, may be easily detected from radiographs (Fig. 3). We tried to control for this potential bias mathematically. We hypothesized that such accurate measurements from radiographs could even be considered "gold standard" measurements for the 2D:4D index.

Our material was relatively large and the repeatability of measurements excellent. Any major bias in the measurements seems improbable. Our average radiographic index (0.925) was somewhat lower than that (1.00) reported in a study of 400 females, whose index was directly measured [2].

Instead of only computing associations between single variables and the 2D:4D index we also used cluster analysis to form two clusters in each of the dimensions representing anthropometric, behavioural, nutritional, work-, health- and fertility-related features. Such clusters consist of individuals that resemble each other in terms of items represented in the cluster. We thus explored a wide range of human features both individually and as features combined in meaningful categories. Several single variables in our study may be related to direct or indirect hormonal effects.

Finding no association between the radiographic 2D:4D index and the studied items suggests that the possible importance of the index, if present, is mostly dependent on the soft tissues of fingers and not on their bony parts. There are two major differences between radiographic and direct finger measurements: soft tissues on fingertips and at the base of fingers. The former seems to be relatively constant from finger to finger *per se *and accounts for a few extra millimetres in fingers. The base of finger measured from the palmar wrinkle differs 1-2 cm from the more proximal origin of the base of the proximal phalanx and cannot exactly be measured from radiographs. Due to the lacking associations between bony measurements and the studied items this soft tissue should therefore be fundamental; otherwise the value of the index as a whole may be questionable.

Hand embryogenesis is a very complex procedure [17]: the growth of the autopod as whole including the metacarpals and finger bones is controlled by the HOX gene system. A protein called Sonic hedgehog specifies normal digit identities and also regulates the expression of the 5' HOXD genes. The apoptosis process (signalled by BMP proteins, which in turn are dependent on the Sonic hedgehog) works to separate the fingers from each other. It seems that the HOX gene system controls the growths of both the bony and probably also the soft tissue parts of fingers. Therefore, both the bony and soft tissue 2D:4D indices could be expected to behave similarly and finding no association between bony finger lengths and the studied variables suggests that there probably is no relation between finger soft tissue and the studied variables either.

Concerning the 2D:4D index mostly positive associations with sexual, behavioural and other variables have previously been published. This may be due to a publication bias, however. A large study of young, normal Danish men failed to show any reliable association between 2D:4D finger ratio and testicular function [18].

## Conclusion

We failed to demonstrate meaningful relations between the radiographic 2D:4D index and the wide scale of studied variables. Despite the ideal set-up of the measuring possibilities in a relatively large radiographic material the variables currently studied were not dependent on the length of finger bones. It can therefore be questioned whether any real associations between the bony 2D:4D index in adult life and (direct or indirect) hormone dependent effects exist. There may be a publication bias explaining that mostly positive findings have been previously reported. This study cannot rule out the possibility that the previously found associations between the 2D:4D index and several items are due to finger soft tissues.

## Competing interests

The author(s) declare that they have no competing interests.

## Authors' contributions

TV conceived and designed the study, performed statistical analyses other than cluster analyses and mainly drafted the manuscript. SS collected the clinical data, designed and performed the cluster analysis. PL-A participated in the design and coordination of the study. All authors participated in drafting, reading, revising and approved the final manuscript.
